# Detection of Quorum Sensing Signal Molecules and Identification of an Autoinducer Synthase Gene among Biofilm Forming Clinical Isolates of *Acinetobacter* spp.

**DOI:** 10.1371/journal.pone.0036696

**Published:** 2012-07-16

**Authors:** Deepa Anbazhagan, Marzida Mansor, Gracie Ong Siok Yan, Mohd Yasim Md Yusof, Hamimah Hassan, Shamala Devi Sekaran

**Affiliations:** 1 Department of Medical Microbiology, Faculty of Medicine, University of Malaya, Kuala Lumpur, Malaysia; 2 Department of Anaesthesiology, Faculty of Medicine, University of Malaya, Kuala Lumpur, Malaysia; University of Florida, United States of America

## Abstract

**Background:**

Quorum sensing is a term that describes an environmental sensing system that allows bacteria to monitor their own population density which contributes significantly to the size and development of the biofilm. Many gram negative bacteria use *N-*acyl-homoserine lactones as quorum sensing signal molecules. In this study, we sought to find out if the biofilm formation among clinical isolates of *Acinetobacter* spp. is under the control of autoinducing quorum sensing molecules.

**Methodology/Principal Findings:**

Biofilm formation among clinical isolates of *Acinetobacter* spp. was assessed and the production of signal molecules were detected with *Chromobacterium violaceum* CV026 biosensor system. Characterisation of autoinducers was carried out by mass spectrometric analysis. We have also reported the identification of an autoinducer synthase gene, *abaΙ* among the isolates that produce quorum sensing signal molecules and have reported that the mutation in the *abaI* gene influences their biofilm forming capabilities. Using a microtitre-plate assay it was shown that 60% of the 50 *Acinetobacter* spp. isolates significantly formed biofilms. Further detection with the biosensor strain showed that some of these isolates produced long chain signal molecules. Mass spectrometric analysis revealed that five of these isolates produced *N-*decanoyl homoserine lactone and two isolates produced acyl-homoserine lactone with a chain length equal to C_12_. The *abaΙ* gene was identified and a tetracycline mutant of the *abaΙ* gene was created and the inhibition in biofilm formation in the mutant was shown.

**Conclusions/Significance:**

These data are of great significance as the signal molecules aid in biofilm formation which in turn confer various properties of pathogenicity to the clinical isolates including drug resistance. The use of quorum sensing signal blockers to attenuate bacterial pathogenicity is therefore highly attractive, particularly with respect to the emergence of multi antibiotic resistant bacteria.

## Introduction

*Acinetobacter* spp. are gram negative aerobic coccobacilli that are ubiquitous in nature, persistent in the hospital environment, and cause a variety of opportunistic nosocomial infections [1]. Among the *Acinetobacter* genomic species, *Acinetobacter baumannii* is recognized as the species most frequently isolated from patients. *Acinetobacter* spp. have been isolated from various types of opportunistic infections, including septicemia, pneumonia, endocarditis, meningitis, skin and wound infection, and urinary tract infection [1]. The distribution by site of *Acinetobacter* infection does not differ from that of other nosocomial Gram-negative bacteria. Often, *Acinetobacter* spp. emerged as important pathogens in the intensive care unit (ICU) setting, and this is probably due to the increasingly invasive diagnostic and therapeutic procedures used in hospital ICUs [2]. As it is a multi-drug resistant organism, infections are difficult to treat [3], resulting in mortalities of 23% for hospitalised patients and 43% for patients under intensive care [4]. The Antimicrobial Availability Task Force of the infectious Diseases Society of America recently identified *A. baumannii*, along with *Aspergillus* spp., extended–spectrum β-lactamase producing *Enterobacteriaceae*, Vancomycin-resistant *Enterococcus faecium*, *Pseudomonas aeruginosa* and methicillin-resistant *Staphylococcus aureus*, as ‘particularly problematic pathogens’ for which there is a desperate need for new drug development [5]. Similarly, the SENTRY Antimicrobial Surveillance Program lists *Acinetobacter* spp. as the causative agent in 2.3 to 3.0% of health care-associated pneumonia and as the eighth most common pathogen (4.0%) isolated from ICU patients worldwide [6]. Thus, *Acinetobacter* spp. is emerging as an increasingly important multidrug resistant pathogen, spreading in hospitals, and causing severe adverse outcomes. Besides that, *Acinetobacter* spp. seems to be spreading from hospital to hospital, and it has caused endemic infections in various geographical areas through multiple hospital outbreaks. It has become a leading nosocomial pathogen in many hospitals as compared to other non-fermenting Gram-negative bacilli. Therefore, a new strategy in the successful treatment of *Acinetobacter* infections is an absolute necessity.

Most *Acinetobacter* spp. research to date has focused on cataloguing and understanding the variety of antimicrobial resistance genes and mechanisms found within the species [7], [8], [9], [10]. It has been shown that *A. baumannii* forms biofilm with enhanced antibiotic resistance [11], [12]. Biofilm formation is a trait closely related to pathogenicity. Bacteria form multicellular biofilm communities on most surfaces. Formation of these sessile communities and their inherent resistance to antimicrobial agents are at the root of many persistent and chronic bacterial infections. Biofilms represent microbial societies with their own defense and communication systems. According to a public announcement from the US National Institutes of Health more than 80% of all microbial infections involve biofilms [13], [14].

Very little is known regarding factors required for biofilm formation in *A. baumannii*. A chaperone and a protein that are required for this process has been identified [15], [16]. The expression of an operon, *pga*ABCD, encoding a poly *N-*acetyl glucosamine (PGA) extracellular matrix has been shown to have a role in biofilm formation [17], [18].

There are reports suggesting that the autoinducer (AI), quorum sensing (QS) signal molecules play an important role in biofilm formation [19], [20]. Studies on two fundamental bacterial small-molecule signaling pathways: extracellular quorum-sensing signaling and intracellular cyclic dinucleotide signalling suggested how these two pathways may converge to control complex processes including multicellularity, biofilm formation, and virulence [19].

Cell-to-cell signalling is often mediated by the production of *N-*acyl-homoserine lactone (AHL) signalling molecules [21]. These are synthesized by the family of homologue proteins. They use the appropriately charged acyl carrier protein as the major acyl chain donor and *S*-adenosyl methionine, which provides the homoserine moiety [22]. To detect these signals, various biosensor strains have been constructed [23], [24], [25]. These biosensors have been used previously to show AHL productions in *Acinetobacter* spp. By using an *Agrobacterium tumefaciens* reporter strain it was shown that all the cultures produced two to four detectable signal molecules with different chromatographic patterns. In *A. calcoaceticus* BD413 supernatants four compounds were detected in a time-dependent manner, and maximal activity was reached at stationary phase [26]. There is a previous report on the AHLs produced by *Acinetobacter baumanii* and it has been shown that mutations in autoinducer synthesis lead to the formation of biofilms with abnormal structures in *Acinetobacter baumannii* [27].

In this study, we report the detection of AHL signal molecules among the 50 biofilm forming clinical isolates of *Acinetobacter* spp. from University of Malaya Medical Centre, Malaysia using the *Chromobacterium violaceum* CV026 biosensor monitor system. Mass spectrometry was used to identify the AHL signals produced by these isolates. An autoinducer synthase designated *abaI* has also been identified among these clinical isolates producing QS signal molecules, a mutant of this gene has been created and has been shown to influence the biofilm forming capabilities. The impairment in the biofilm formation was rescued by ethyl acetate extracts of the culture supernatants from the wildtype strain. These findings could allow further investigations into the antagonists of the QS signal molecules, which could inhibit biofilm formation among the clinical pathogens.

## Results

### Quantification of Biofilm Formation Using Microtiter Plate Method

**Figure 1:**
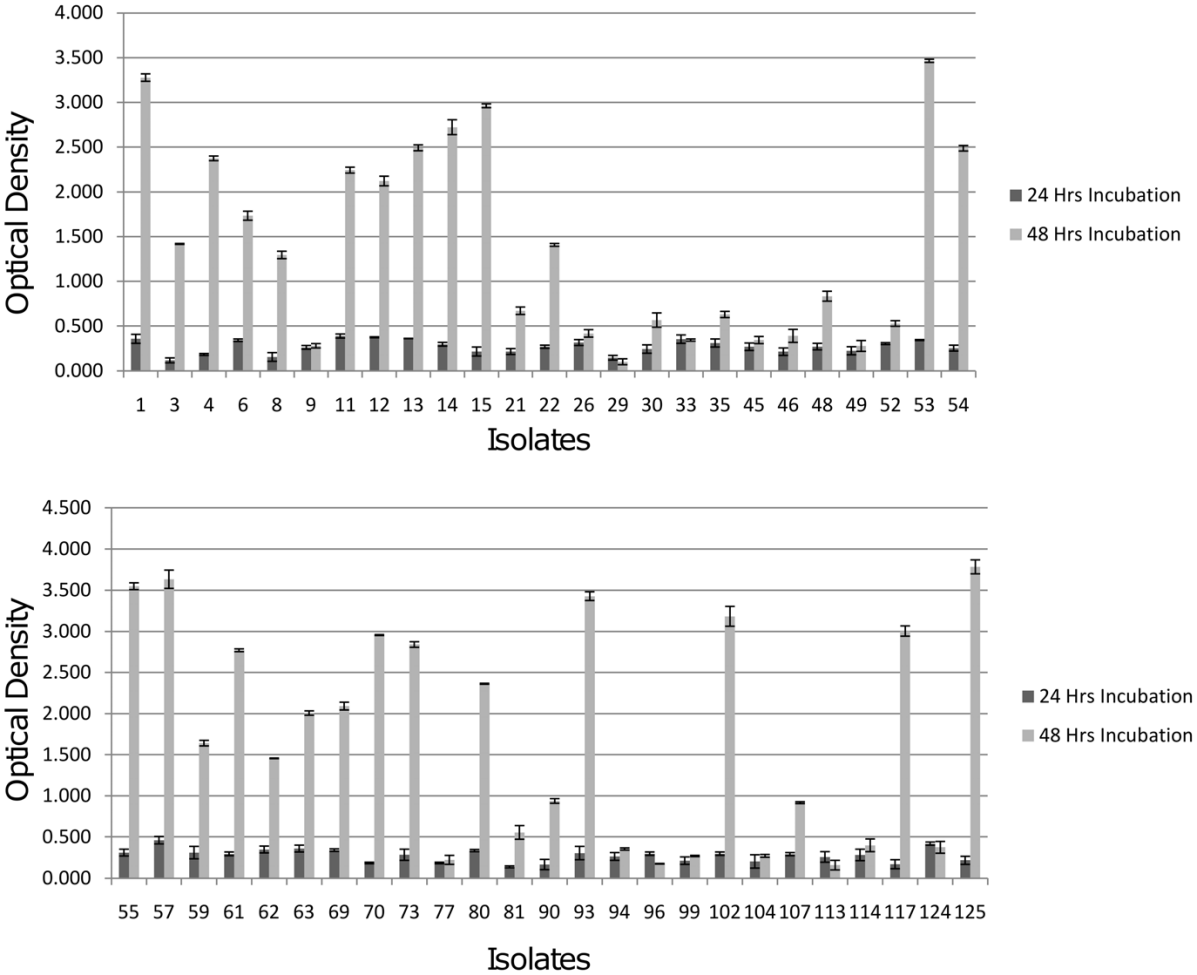
Biofilm formation among the 50 isolates of *Acinetobacter* spp. in microtiter plates. Strain numbers are designated in x axis. About 60% of the isolates significantly forms biofilms when incubated for prolonged hours. Data are pooled from triplicate experiments. The results are expressed as the mean and the error bars indicate the standard deviation. The *P* value was less than 0.05 (*P*<0.05).

Quantification of biofilm produced by the 50 clinical isolates of *Acinetobacter* spp. revealed that 30(60%) isolates significantly formed biofilms under prolonged period of incubation (*p* value <0.05). Figure 1 shows the graphical representation of the biofilm formation among all the 50 isolates. Quantification of biofilm formation after prolonged incubation periods showed that in all the 50 isolates 48 hrs incubation showed higher biofilm formation compared to 24 hrs of incubation (Fig. 1). Isolates with no change in OD over the control were classified as non-biofilm formers. The microtitre plate assay was done in triplicates and the data were pooled from these experiments. Thus the graph also shows the standard deviation.

### Screening for AHLs Among the Isolates

**Figure 2:**
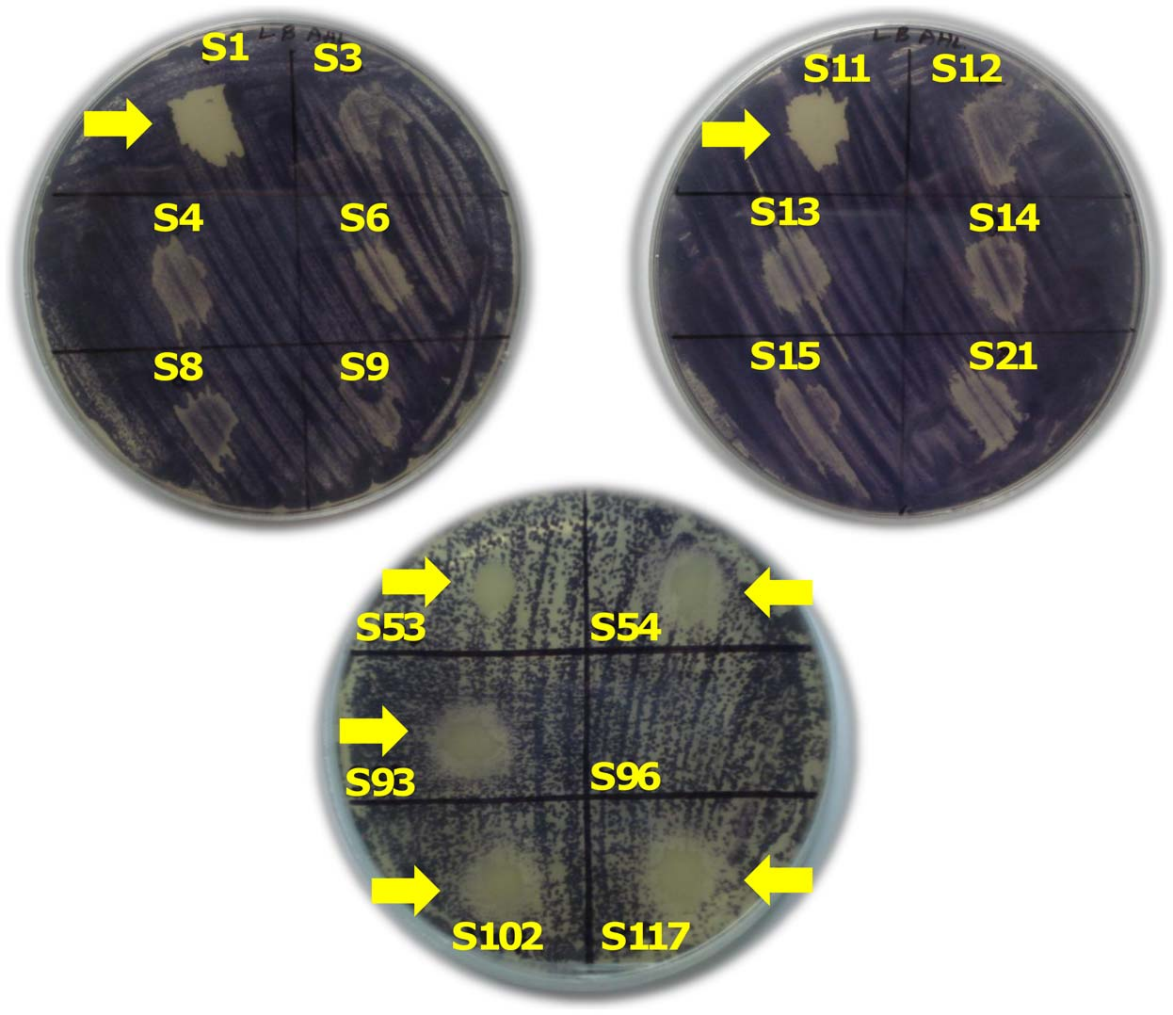
Agar plate assays for screening the production of acylated homoserine lactone (AHL) in *Acinetobacter* spp. clinical isolates using *Chromobacterium violaceum* CV026 (Inhibition) as monitor system. All the seven isolates that tested positive for long-chain AHLs are shown.

When CV026 induction test was carried out for the detection of short chain AHLs, all the 50 isolates produced colourless colonies. This showed that none of these *Acinetobacter* spp. clinical isolates produced short chain AHL molecules. In CV026 inhibition test, 7 of the isolates marked as S1, S11, S53, S54, S93, S102, S117 tested positive for long- chain AHL production. They produced colourless colonies in the induced chromoplate, while the negative isolates produced purple colonies (Fig. 2). The CV026 induction test was done on the chromoplate and the positive colonies producing the AHLs are expected to produce purple colonies. None of the tested isolates were positive for the induction test. CV026 inhibition test was carried in a chromoplate to which the synthetic AHL was previously added. Here long-chain AHLs are expected to inhibit the induced CV026 and thus produce colourless colonies in the purple background. Seven isolates tested positive for long-chain AHLs.

### Quantification of the AHLs by Well-diffusion Assay

**Figure 3:**
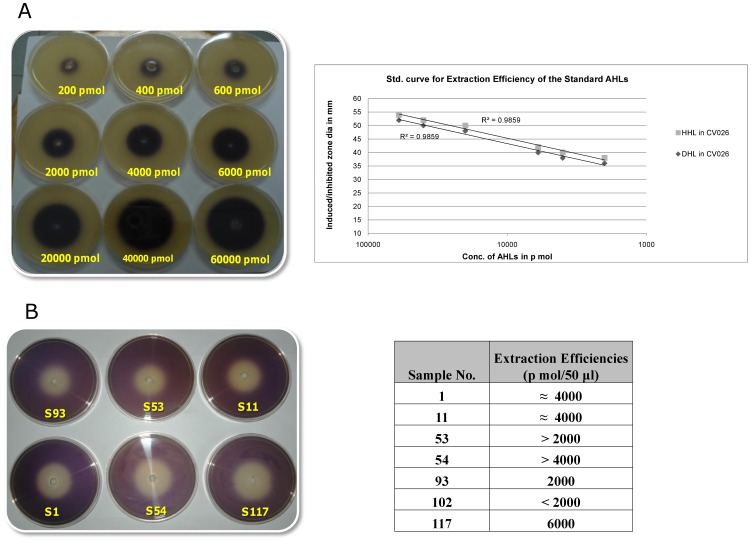
(A) *Chromobacterium violaceum* **CV026 well-diffusion assay in LB agar**. *N-*hexanoyl homoserine lactone (HHL) was added to wells in agar containing *Chromobacterium violaceum* CV026. Zones of HHL-induced violacin production is seen surrounding the wells. Increasing concentration of AHL shows the increase in zone diameters. Standard curve showing the relationship between concentration of acylated homoserine lactone (*N-*hexanoyl homoserine lactone (HHL) & *N-*Decanoyl homoserine lactone (DHL)) and resulting diameters of induced/inhibited zones in *Chromobacterium violaceum* CV026 monitor system. (B) Well-diffusion assay for the tested isolates. Extraction efficiencies were tested on supernatants from *Acinetobacter* strains. Yields obtained were in the range of 1×10^−9^ moles to 6×10^−9^ moles of AHLs.

AHLs were extracted and quantified by the well diffusion assay. Increasing amounts of AHL added to the wells in the well-diffusion assay caused increase in the size of induced and inhibited zones surrounding the wells in *Chromobacterium violaceum* CV026 (Fig. 3A). Standard curves were created by comparing log (amount of AHL) to surface area of the zones (Fig. 3A). Based on the standard curves, extraction efficiencies were calculated using DHL (Decanyl Homoserine Lactone) in case of long-chain AHLs and HHL (3-*N-*Hexanoyl homoserine lactone) in case of short-chain AHLs, using both acidified and non-acidified ethyl acetate. Approximately twice as much AHL was extracted when using acidified ethyl acetate as compared to non-acidified. Extraction efficiencies were tested on supernatants from *Acinetobacter* strains and it was observed that acidified extractions gave better yields as compared to the non-acidified extractions. Yields obtained were in the range of 1×10^−9^ moles to 6×10^−9^ moles of AHLs (Fig. 3B). These were later confirmed by quantification through mass spectrometry.

### Mass Spectrometry and Structural Identification of the AHLs

**Figure 4:**
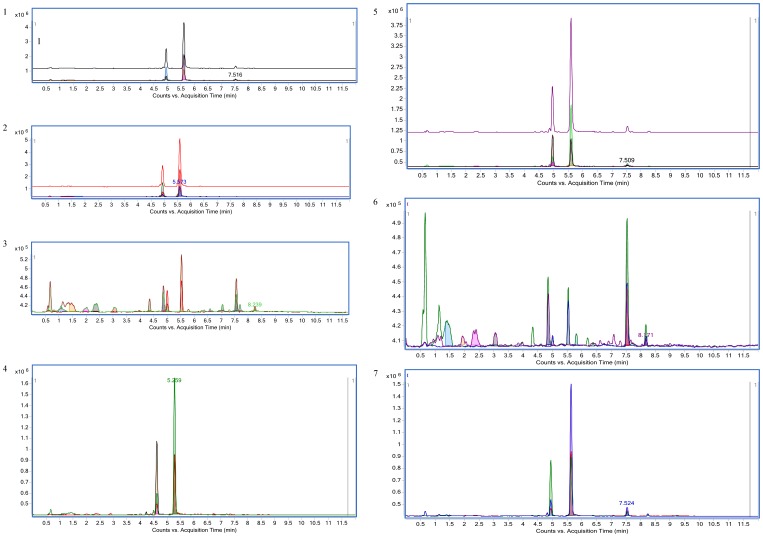
MS profiles of AHL signals. An Agilent 6520 LC-ESI-QTOF in full scanning positive ion mode or auto-MS/MS mode in a range of 50–1600 *m/z* was used. TIC trace of each sample (1, 11, 53, 54, 93, 102, 117) with overlaid common precursor ions are respectively shown in figures 1, 2, 3, 4, 5, 6 and 7. Each of the peaks was then analyzed by MS to identify the parent ion, and the full MS profile for each ion was established to identify the exact chain lengths of the AHLs.

Mass spectrometric analysis revealed the presence of C_10_ AHLs in the tested 5 isolates (S1, S11, S54, S93 and S117) and other 2 isolates (S53, S102) produced AHLs with chain lengths C_12._ Of the masses that were reported the only ones that seem to make sense are 284 and 282 as an AHL with a 12 carbon acyl chain, with the lower mass (282) containing a single alkene (Table 1). An alkene could be anywhere along the chain length, however it can be rationalized that it could be a dehydration product from a beta-hydroxy derivative. The retention time at a later time than the decanoyl standard fits well. These masses were found in samples 53 and 102, which well confirms the presence of chain lengths C_12_. The TIC trace of each sample (1, 11, 53, 54, 93, 102, 117) with overlaid common precursor ions are shown in Fig. 4.

**Table 1:** Observed precursors displaying fragments corresponding to 102.1, 74.1 and 71.1.

Sample	Compound	Mass	Retentiontime (min)	Fragments
**1**	1	274.7	4.94	102.1
		274.7	4.94	74.1
		274.6	4.94	71.1
	2	256.7	5.59	102.1
		256.7	5.59	74.1
		256.7	5.59	71.1
	3	256.7	7.51	102.1
**11**	1	317.6	1.36	102.1
	2	274.7	4.92	102.1
		274.8		74.1
		274.8		71.1
	3	256.6	5.57	102.1
		256.7		74.1
		256.7		71.1
**53**	1	317.7	1.45	102.1
	2	212.6	2.37	71.1
	3	246.6	3.02	71.1
	4	471.1	4.34	102.1
	5	274.8	4.85	102.1
		274.7		71.1
	6	318.8	4.99	74.1
	7	300.1	5.51	74.1
	8	256.9	7.54	74.1
		256.7		102.1
		256.8		71.1
	9	282.8	7.68	71.1
	10	284.6	8.23	102.1
**54**	1	274.7	4.59	102.1
		274.8		74.1
		274.8		71.1
	2	256.8	5.26	74.1
		256.8	5.26	71.1
		256.8	5.26	102.1
**Sample**	**Compound**	**Mass**	**Retention time (min)**	**Fragments**
**93**	1	274.7	4.59	102.1
		274.7		71.1
		274.7		74.1
	2	256.7	5.58	102.1
		256.7		71.1
		256.7		74.1
	3	256.6	7.51	74.1
**102**	1	317.6	1.4	102.1
	2	212.5	1.93	71.1
	3	212.5	2.39	71.1
	4	246.7	3.04	71.1
	5	274.8	4.83	102.1
	6	318.8	4.98	74.1
	7	301	5.58	74.1
	8	363.1	5.79	102.1
	9	256.8	7.52	71.1
		256.8		102.1
		256.8		74.1
	10	284.7	8.16	102.1
		284.6		74.1
		284.9		71.1
**117**	1	274.7	4.92	71.1
		274.8		74.1
		274.7		102.1
	2	256.7	5.61	71.1
		256.7		74.1
		256.7		102.1
	3	256.7	7.52	71.1
		256.7		74.1
		256.7		102.1

### Identification of *abaI* Gene in *Acinetobacter* spp. Producing the QS Signal Molecules

**Figure 5:**
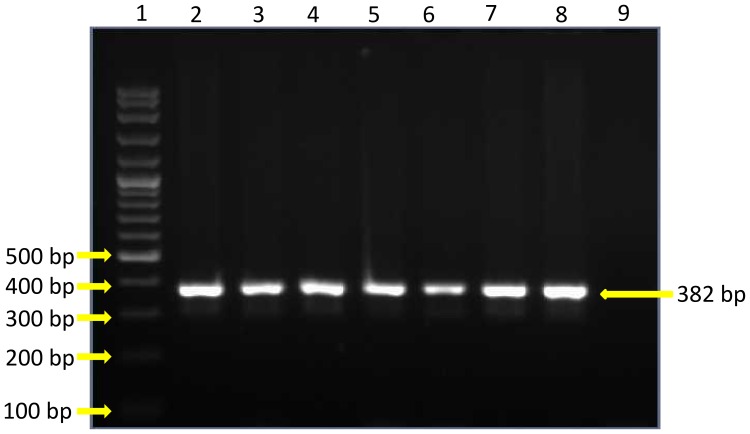
The electrophoretic banding patterns of the PCR products using the primers for the *abaI* gene. Lane 1–100bp DNA ladder, lane 2, 3, 4, 5, 6, 7, 8– amplicons from isolates S1, S11, S53, S54, S93, S102 and S117 respectively, lane 9– Negative control.

Polymerase Chain Reaction (PCR) for *abaI* gene, produced amplicons of 382 bp in all the 7 isolates (isolate no.S1, S11, S53, S54, S93, S102, S117) which produced the QS signal molecules. The electrophoretic banding patterns are shown (Fig. 5).

### Organisation of the *abaI* Region in *Acinetobacter* spp. (S117)

The amplicon of the *abaI* gene from isolate S117 was further sequenced and analysed. Isolate S117 showed the maximum differences in the nucleotides and thus the aminoacid sequence of the autoinducer synthase protein when compared with the already reported *abaI* gene sequences. Hence this protein was chosen for all subsequent experiments. The BLAST analysis program revealed that the *abaI* gene identified in *Acinetobacter* spp. isolate S117 is 92% identical to the corresponding protein reported in recently published *A. baumannii* ATCC 17978 genome [28]. The AbaI protein shared 91% identity and 97% similarity with the already reported AbaI protein of *A. baumannii* M2 strain [27]. Further, the BLAST analysis also revealed that the AbaI protein exhibited 61% identity and 79% similarity to the acyl homoserine lactone synthase of *Halothiobacillus neapolitanus* c2, 65% identity and 75% similarity to the autoinducer synthesis protein of *Acidithiobacillus ferrooxidans* ATCC 53993, 47% identity and 67% similarity with autoinducer synthatase family protein of *Burkholderia mallei* ATCC 23344 and 49% identity and 63% similarity with *N-*acyl homoserine lactone synthase from *Pseudomonas fluorescens*. This sequence has already been submitted to the NCBI Genbank (Accession number HQ013310).

### Mutation in the *abaI* Gene

**Figure 6:**
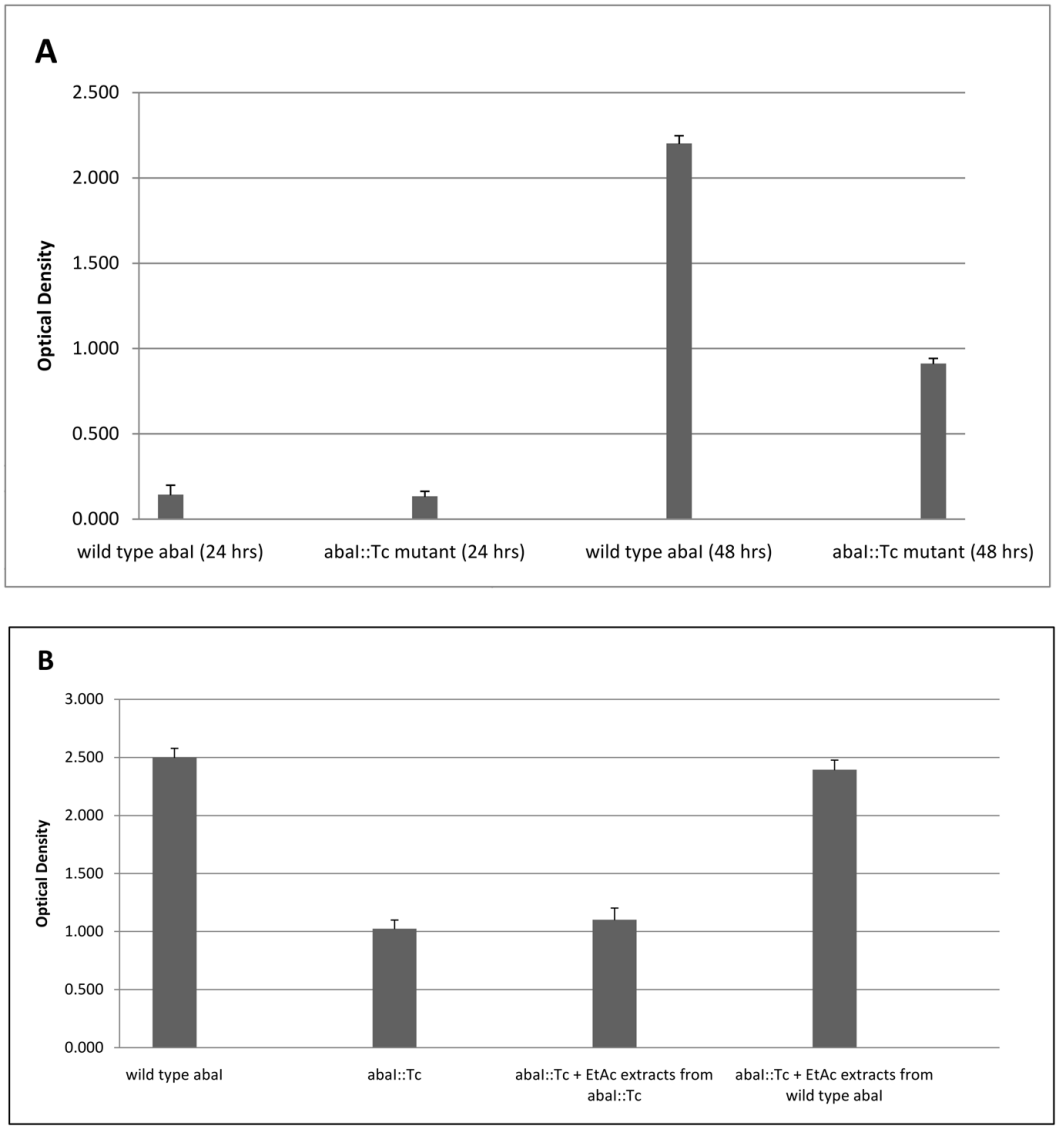
Biofilm formation of the wild type *abaI* and *abaI*::Tc mutant. (A) The microtiter plate was employed to evaluate the biofilm formation among the wild type *abaI* and *abaI*::Tc mutant over time. There is considerable inhibition of biofilm formation in the *abaI*::Tc mutant compared to the wild type. (B) Ethyl-acetate (EtAc) extracts of wild type and the *abaI*::Tc mutant from the culture supernatants were added for the complementation experiments.

The *abaI* gene from isolate S117 cloned into plasmid vector pCR 2.1-TOPO and transformed into chemically competenet *E.coli* cells. These cells carrying the wild type *abaI* gene was found to produce the same kind of AHL signal produced by S117 in the thin layer chromatography bioassay overlay (results not shown). The *abaI*::Tc mutant was further constructed which failed to produce any detectable signals using the biosensor strain (results not shown). The examination of the ability of the wild type and the *abaI*::Tc mutant to form biofilms showed that there was inhibition in the biofilm formation in the *abaI*::Tc mutant when compared to the wild type (Fig. 6A). The mutation complementation experiment with the ethyl-acetate extracts of the culture supernatants from wild type and the *abaI*::Tc mutants showed that the addition of ethyl-acetate extracts from the wild type cells restored the ability of the *abaI*::Tc mutants to form normal biofilms. But, the ethyl-acetate extracts from the *abaI*::Tc mutants failed to restore the biofilm forming capabilities in the *abaI*::Tc mutant (Fig. 6B).

## Discussion

The current view of biofilm infections leads to the realization that their effective control will require a concerted effort to develop therapeutic agents that target the biofilm phenotype and community signalling–based agents that prevent the formation, or promote the detachment, of biofilms [29]. Several molecules have been associated with the coordination of activities of microorganisms within a community. These include AHL, oligopeptides, amino acids such as glutamate and aspartate, and fatty methyl esters. It is conceivable that one or more of these molecular signals may impact biofilm physiology [15], [30].

QS is a regulatory mechanism which enables bacteria to make collective decisions with respect to the expression of a specific set of genes. These may include genes involved in biofilm formation and virulence. They play a key role in orchestrating the expression of exoproteases, siderophores, exotoxins and several secondary metabolites, and participate in the development of biofilms.

It has been shown that in contrast to the other QS systems, the AHL-mediated QS signalling system appears to control genes essential for colonisation of eukaryotes across a large number of bacterial species. This process is facilitated by bacterial biofilms [29]. The high density of bacteria within biofilms has led to the speculation that quorum-sensing genes and AHL production may be fundamentally associated with biofilm physiology. It has been shown that mutations in autoinducer synthesis lead to the formation of biofilms with abnormal structures in *Acinetobacter baumannii* [27].

In our study, it is observed that about 60% of our clinical isolates of *Acinetobacter* spp. significantly formed biofilms under prolonged period of incubation. When investigations on the sources of isolation of these isolates were done, they were equally from the upper and lower respiratory tracts. All these isolates were also screened for the QS signal molecule production and 7 of these isolates effectively produced long chain AHL molecules by using the CV026 biosensor monitor system. On analysis of the biofilm forming capabilities of the 7 isolates that produced the long chain AHls, they all form biofilms significantly. The method presented here for screening pure cultures of *Acinetobacter* spp. for AHL production using the *Chromobacterium violaceum* CV026 monitor system has not been used previously. It is rapid and does not require sterile supernatants or AHL extractions from cultures. It also has the advantages of only requiring monitor strains and the ability of the strains tested to grow on LB media. Also about 6 strains can be tested on the same plate. The only limitation of this assay is that the detection limit lies within the scope of the signal monitor system CV026 that was used and this monitor system is poor in sensing some of the signal molecules.

In this study, it has been shown that among the 30 biofilm forming isolates, only 7 produced long-chain AHLs. This may be due to the fact that biofilm formation is a multifactorial event and QS signals are one of such factors. Genetic analysis of biofilm formation has led to the proposal that extracellular signals and quorum sensing regulatory systems are essential for differentiated biofilms. Hence, differentiated biofilms may also be the net result of many independent interactions, rather than being determined by a particular global Quorum Sensing system [31].

Further mass spectrometric analysis confirmed the presence of C_10_ AHLs in the tested 5 isolates and other 2 isolates produced AHLs with chain lengths C_12._ Of the masses that were reported the only ones that seem to make sense are 284 and 282 as an AHL with a 12 carbon acyl chain, with the lower mass (282) containing a single alkene (Table 1). An alkene could be anywhere along the chain length, however it can be rationalized that it could be a dehydration product from a beta-hydroxy derivative. The retention time at a later time than the decanoyl standard fits well. These masses were found in samples 53 and 102. In Table 1, it is shown that some strains produce a signal with an m/z of 274 that gives rise to a MS/MS product of 102 suggesting it is likely comprised of an HSL moiety. This could be due to the presence of minor quantity of 3-oxo- C11 HSLs. However, none of the clinical isolates tested produced 3-OH-C12 seen in the M2 strain described by Niu *et al* [27].

Further quantification of the QS molecules by well diffusion assay revealed that a minimum of approximately 2×10^−9^ moles and a maximum of 6×10^−9^ moles of AHLs were detected among the clinical isolates of *Acinetobacter*. Quantifications done by mass spectrometric analysis also confirms these values.

An autoinducer synthase gene in *A. baumannii* that was designated as *abaI* has already been identified and characterised. The deduced AbaI protein was highly similar to the members of the LuxI family. It has been shown that this autoinducer synthase was required for normal biofilm development [27]. In our study we have identified and analysed *abaI* gene which encodes a similar protein. The BLAST analysis revealed that the *abaI* protein has only 91% identity with the already reported protein, but still retains its function as a quorum sensing autoinducer synthase. Further, the similarity of the identified AbaI protein from S117 to various species of *Burkholderia*, *Pseudomonas fluorescens* and *Acidithiobacillus ferrooxidans* suggests a common origin of these genes.

In this study we have further cloned the *abaI* gene. The cloned gene was further mutated and a *abaI*::Tc mutant of the gene was created and the mutant was shown to have inhibition in the biofilm formation. This shows that the *abaI* gene product was required for biofilm formation (Fig. 6). The differences between the wild type and the *abaI*::Tc mutant clearly showed that *abaI* directed AHL pathway is required for efficient biofilm formation. The mutation complementation experiments reveal that the wild type phenotype can be restored.

This study has revealed that it is likely that QS regulation is important for biofilm development, but there is no reason to assume that this type of regulation is the only important effector. In a given setting, the biofilm associated community will exploit all available adaptive mechanisms and the corresponding network of regulatory activities (including QS) and it is not possible to unequivocally assign a specific determining regulatory factor for the structure/function relationship of the community.

In conclusion, the use of simple methods for detection of AHLs and their involvement in biofilm formation have been presented. These methods can be used to determine to certain extent the type of AHLs produced as well as to study the increase in AHL concentration over time. Mass spectrometric analysis revealed the exact structure of these AHL molecules to be C10 and C12.

The identification of the *abaI* and the AHL signals will allow the identification of the antagonists that inhibit biofilm development. These antagonists may reduce the ability of *Acinetobacter* spp. to survive on environmental surfaces for extended periods, a key component of its ability to form biofilms and persistence in intensive care wards.

## Materials and Methods

### Bacterial Strains and Culture Conditions

Fifty strains that were identified as *Acinetobacter* spp. were obtained from the Diagnostic Bacteriology Laboratory, University Malaya Medical Centre (UMMC) between August 2003 to March 2004. The strains were obtained from invasive and non-invasive sites which include blood, tracheal secretion, sputum, throat swab, peritoneal fluid, wound, bronchial lavage, and urine. The details of the source of isolation of all these isolates have been listed (Table 2). The identity of the isolates was confirmed using standard laboratory methods which include Gram-stain, colony morphology, lactose fermention, and reaction to the oxidase test [32]. The isolates were further reconfirmed to belong to the *Acinetobacter baumannii/calcoaceticus* complex using API20NE kit (bioMerieux, France). These isolates were all screened for the production of quorum sensing signal molecules. The test isolates were grown on LB (Difco).

**Table 2:** Strain number, patient number, ward, source and date of isolation of 50 *Acinetobacter* spp. isolates.

No.	Patient No.	Date Collected	Ward	Source
**1**	1106728	29/08/2003	ICU	Tracheal aspiration
**11**	1147833	03/12/2003	8U	Tracheal
**49**	1163189	07/01/2004	13U	Sputum
**99**	1176996	09/02/2004	13U	Sputum
**9**	11464697	01/12/2003	6TE	Tracheal secretion
**21**	1152147	12/12/2003	ICU	Tracheal secretion
**93**	1170717	25/01/2004	ICU	Tracheal secretion
**102**	1179797	14/02/2004	7U	Wound swab
**3**	1133309	28/10/2003	6TD	Blood-PICC
**4**	1137968	07/11/2003	6TD	Blood C+S
**8**	1146957	02/12/2003	P2	Blood
**70**	1166501	14/01/2004	P5	Throat swab
**113**	1186526	01/03/2004	8D	Throat swab
**117**	1188198	04/03/2004	ICU	BAL
**6**	1147974	04/12/2003	6U	Tracheal secretion
**12**	1149607	08/12/2003	CICU	Tracheal secretion
**13**	1149580	08/12/2003	8D	Wound swab
**14**	1149626	08/12/2003	ICU	Tracheal secretion
**15**	1150413	09/12/2003	ICU	Tracheal secretion
**22**	1151037	10/12/2003	CICU	Wound swab
**26**	1155939	21/12/2003	P1	Peritoneal fluid
**29**	1157633	24/12/2003	ICU	Tip for epidermal catheter
**30**	1156504	22/12/2003	ICU	Tracheal Secretion
**33**	1157503	24/12/2003	7U	BAL
**35**	1157917	26/12/2003	6U	Tracheal secretion
**45**	1159599	30/12/2003	6U	Abrasion over back
**46**	1160751	01/01/2004	ICU	Tracheal secretion
**48**	1163171	07/01/2004	7U	Tracheal secretion
**52**	1162203	05/01/2004	7U	Sputum
**53**	1161971	05/01/2004	8U	Suture line swab
**54**	1164238	09/01/2004	P5	Urine
**55**	1161540	03/01/2004	6U	Wound swab
**57**	1164572	09/01/2004	6U	Tracheal secretion
**59**	1165166	11/01/2004	7U	Bronchial lavage
**61**	1162426	06/01/2004	ICU	Tracheal secretion
**62**	1161661	04/01/2004	13U	Tracheal secretion
**63**	1162120	05/01/2004	P1	Bronchial lavage
**69**	1167282	15/01/2004	7U	Tracheal secretion
**73**	1168513	18/01/2004	7U	Bronchial 3 plugged & mucous
**77**	1167614	15/01/2004	ICU	Peritoneal fluid
**80**	1171825	27/01/2004	13U	Tracheal secretion
**81**	1171359	26/01/2004	13U	Swab & foot pressure sore
**90**	1171487	26/01/2004	ICU	Tracheal secretion
**94**	1176566	08/02/2004	6U	Pus swab
**96**	1176799	09/02/2004	7D	Wound Swab
**104**	1177833	10/02/2004	ICU	Double lumen tips
**107**	1180637	16/02/2004	12U	Wound swab
**114**	1186122	28/02/2004	12U	Sputum
**124**	1184548	25/02/2004	13U	Tracheal aspiration
**125**	1185678	26/02/2004	ICU	Tracheal secretion

The biosensor strains detect AHL signals by the activation of a reporter gene such as lacZ or lux or by the production of or inhibition of a purple pigment in *Chromobacterium violaceum*. *Chromobacterium violaceum*, a Gram-negative bacterium commonly found in soil and water, produces the characteristic purple pigment violacein. Previously the authors described a violacein-negative, mini-Tn5 mutant of *Chromobacterium violaceum* (CV026) in which pigment production can be restored by incubation with supernatants from the wild-type strain. This mutant was developed to be used as a general biosensor for AHLs. In CV026, violacein is inducible by all the AHL compounds evaluated with *N-*acyl side chains from C_4_ to C_8_ in length, with varying degrees of sensitivity. Although AHL compounds with *N-*acyl side chains from C_10_ to C_14_ are unable to induce violacein production, if an activating AHL is incorporated into the agar, these long-chain AHLs can be detected by their ability to inhibit violacein production. *Chromobacterium violaceum* strain CV026 was the monitor strain used to screen for AHL-producing *Acinetobacter* strains. The CviR (*Chromobacterium violaceum* Repressor) of *Chromobacterium violaceum* strain CV026 (Throup *et al*., 1995) [33] regulates the production of a purple pigment when induced by AHL. Certain long-chained AHLs reversibly inhibit the induced CviR resulting in a lack of pigment when compared to a control. The CV026 strain was routinely grown in a shaker incubator in Luria Bertani broth (LB) (1% peptone, 0.5% yeast extract, 0.5% NaCl; PH 7.00) (Bertani, 1951) [34] solidified with 1.25% agar when required and supplemented with 20 µg/µl of kanamycin. For screening AHLs, TY medium containing (per liter) 8 g of Bacto Tryptone (Difco Laboratories), 5 g of yeast extract, and 5 g of NaCl (pH 7) was used.

### Quantification of Biofilm Formation Using Microtiter Plate Method

Biofilm formation was determined by the ability of cells to adhere to the base of 96-well polystyrene plate using the method of Boddey *et al* [35] with modification. Briefly, 100µl of LB broth was added into each well of sterile 96-well polystyrene plate followed by the addition of 1µl of bacterial culture that was grown at 37°C overnight with shaking at 150 rpm. The plate was incubated without shaking at 37°C for 18 hours. Thereafter, 1 µl from each well was transferred into triplicate wells of a fresh 96-well plate containing 100 µl of fresh LB and the plates incubated without shaking for 24 hours at 37°C. Following incubation the supernatant was discarded carefully and the wells were stained with 150µl of 1% crystal violet at room temperature for 30 minutes. The stain was then removed and the wells were washed twice with 175 µl sterile deionized water, before the addition of 175 µl of dimethyl sulfoxide (DMSO) to solubilize the crystal violet. The plate was then read spectrophotometrically at an absorbance wavelength of 570 nm. For each strain the assay was run in triplicate wells and in three independent experiments. Wells containing only the medium was used as control. The same protocol was followed to quantify the biofilm after prolonged incubation at 37°C for 48 hours and then they were compared to the biofilms formed after incubation for 24 hours.

### Statistical Analysis

The significance of the biolfilms formed by the 50 isolates were statistically analysed by using a one-way analysis of variance (ANOVA) with *post hoc* multiple comparisons. The *P* value was less than 0.05. All the statistical tests were performed by SPSS software.

### Screening for AHL

Screening for AHL production among the 50 isolates was done as follows: A fresh plate of *C.violaceum* CV026 was prepared on LB agar plates containing 20 µg/ml kanamycin. This plate was incubated overnight at 37°C. A loopful of cells was taken and resuspended in 10 µl of sterile water and adjusted to an OD _600 nm_ of approximately 0.4. Then 5 ml of this culture was added to 200 ml of cooled TY agar and poured as a thin plate. The test strains were grown separately in 5 ml of TY broth for 24 hours and then diluted to an OD _600 nm_ of 1.0. About 5 µl drops of the test strains were then added to the previously prepared chromoplate and allowed to dry. The tests are done in duplicate and incubated for 2–3 days at 28°C. This test can detect the presence of short chain AHL producing test strains. For detecting the long chain AHLs producing test strains a variant of this test was carried out in which the chromoplate was prepared along with 75 nM concentration of *N-*Hexanoyl Homoserine lactone (HHL) incorporated in the medium. Monitor strains themselves served as negative control and monitor strains in plate containing 75 nM HHL served as positive control. The basic concept of this method was adopted from McClean *et al* 1997 [23]. This method was previously not used for directly screening AHL producing bacteria.

### Extraction of Culture Supernatants for Mass Spectrometry

Extractions for Mass Spectrometry (MS) were prepared as described by Shaw *et al* 1997 [24] with minor modifications. Ten-milliliter volumes of the bacterial cultures were centrifuged and the supernatants were extracted with an equivalent volume of ethyl acetate acidified by supplementing with 0.5% formic acid. The mixture was shaken vigorously for 30 seconds and the two phases were allowed to separate. The shaking was repeated three times before the ethyl acetate containing fraction was removed and another 10 ml fraction was added. The whole extraction process was repeated three times. The combined extracts were dried filtered and evaporated to dryness. Residues were dissolved in 50–100 µl of HPLC–grade ethyl acetate. Comparisons were made of extraction efficiencies using both acidified and non-acidified ethyl acetate.

### Well-diffusion Assays

Quantifications of the AHLs produced by the test strains can be done by well-diffusion assays. This was done as described by Ravn *et al* 2001 [36] with some modifications. The agar plates were prepared as follows: a preculture of *Chromobacterium violaceum* CV026 was grown in LB for 24 hours at 25°C with aeration and 2 ml of the preculture was used to inoculate 100 µl of LB broth. The culture was grown for 24 hours at 25°C with aeration and was poured into 200 ml of LB agar (1.2% agar) containing 20 µg/µl kanamycin maintained at 46°C. The agar culture solution was immediately poured as 20 ml portions in petri dishes. Fifty µl volumes of AHL containing solutions (either the standard or the extraction from culture supernatants) were pipetted into wells (6 mm) punched in the solidified agar. The plates were incubated at 25°C for 48 hours before the diameter of the AHL induced zones surrounding the wells was measured. For the inhibition of the induced CV026, the LB-agar was supplemented with 75 nM *N-*hexanoyl L-homoserine lactone (HHL, Sigma- Aldrich).

### Mass Spectrometry

Extracts were also subjected to Mass Spectrometric analysis. Sample pellets were re-suspended in a mixture of 50% acetonitrile:distilled water 0.1% formic acid (200 µL) initially using a pipette, then sonicated for several minutes. The samples were centrifuged for 5 minutes at maximum rpm and then the supernatant was carefully removed and transferred to a new vial. 10 µL of each sample was transferred to a sample vial with insert and diluted 1 in 5 by addition of 50% acetonitrile, 0.1% formic acid (40 µL). An Agilent 6420 LC-ESI-QQQ using a dynamic multiple reaction monitor (DMRM) was used for quantification. Instrument conditions were: Gas Temperature: 300°C, Gas Flow: 10.5 L/min, Nebulizer: 45 psi, Capillary (positive): 4000V. Samples were injected (2µL) and separated by reverse-phase chromatography on an Agilent Poroshell 120 EC-C18 (2.1×100 mm, 2.7 µm, 600 Bar) column, using the following buffers (100% water/0.1% FA) and (100% ACN/0.1% FA), with temperature maintained at 35°C.

The samples were analysed using an Agilent 6520 LC-ESI-QTOF in full scanning positive ion mode or auto-MS/MS mode in a range of 50–1600 *m/z.* Instrument conditions were: Gas Temperature: 300°C, Gas Flow: 10.5 L/min, Nebulizer: 45 psi, Capillary (positive): 4000V, Fragmentor: 150V, Skimmer 65V. Samples were injected in 1 µL volumes and separated by reverse-phase chromatography on an Agilent Poroshell 120 EC-C18 (2.1×100 mm, 2.7 µm, 600 Bar) column, using the following buffers (100% water/0.1% FA) and (100% ACN/0.1% FA), with temperature maintained at 35°C.

MS/MS Spectra were collected using the following parameters: MS (Full scan) range: 100–1600 *m/z* collected at a rate of 1 spectra/s. Auto MS/MS range: 50–1600 *m/z* collected at rate of 3 spectra/s with a maximum of 5 precursors selected and fragmented using collision energy of 25V. Precursors were excluded after 1 spectra for a period of 30 seconds.

The standards of beta-ketocaproyl, hexanoyl, heptanoyl, octanoyl and decanoyl were diluted using 100% acetonitrile. The diluted standards were mixed to provide a stock solution containing all standards with the following concentrations: 0.1, 0.5, 1, 5, 10, 50, 100, 500, 1000, 5000 and 10,000 micromolar (µM). A standard curve was run between the concentrations and in each case a curve was fit over the appropriate range, with a linear curve attempted in all cases, except where high concentration points were required. For the experiment where concentrated samples were tested a limited concentration range 0.1 µM –100 µM was used, this significantly reduced signal suppression and turnover of the standard curve. Quantification of AHLs in the sample was done using these standard curves.

### Identification of *abaI* Gene in *Acinetobacter* spp. Producing the QS Signal Molecules

The presence of *abaI* gene encoding an autoinducer synthase was identified by polymerase chain reaction (PCR) using already published primers [27], F-5′-GTACAGTCGACGTATTTGTTGAATATTTGGG-3′ and R-5′-CGTACGTCTAGAGTAATGAGTTGTTTTGCGCC-3′. The final PCR amplification reaction mixture contained 5.0 mM PCR buffer, 8.3 mM MgCl_2_, 200 nM dNTP (MBI, Fermentas, Vilnius, Lithuania), 0.3 µM of each primer pair, 2 units of Taq DNA polymerase (MBI, Fermentas) and 3 µl of DNA template in a final volume of 25 µl. The PCR amplification was performed with mycyler thermocycler (BioRad laboratires, Hercules, CA) with the following PCR temperature cycling parameters: Initial denaturation at 94°C for 10 minutes followed by 30 cycles of denaturation at 94°C for 30 seconds, Primer annealing at 66.5°C for 30 seconds, primer extension at 72°C for 1 minute and the final extension at 72°C for 5 minutes. The resulting amplicons of the isolate S117 has been sequenced and it’s organisation in the genome was analysed.

### Mutation in the *abaI* Gene

The *abaI* gene from isolate S117 was further cloned into plasmid vector pCR 2.1-TOPO using the TOPO TA cloning kit (Invitrogen Corporation, California) following the manufacturer’s instructions. The PCR purification of the *abaI* gene was done using the Expin PCR purification kit (GeneAll Biotechnology, Korea) before cloning. The cloned *abaI* gene was disrupted by insertional inactivation using tetracycline gene cassette at the SspΙ site (blunt end ligation) within the *abaI* gene. The insertion of tetracycline gene in the coding region of the *abaI* gene that prevented the production of the AHL signal was identified using the biosensor strain. To inactivate the *abaI* gene in the chromosome of the *Acinetobacter* spp. S117, the insertion that disrupted the *abaI* gene in the middle along with the flanking chromosomal DNA was cloned into pUC18- based suicide delivery vector. This plasmid pUC18.*abaI*::Tc was introduced into *Acinetobacter* spp. S117 by conjugation. *E.coli* K12 carrying the pUC18.*abaI*::Tc plasmid was the donor. Matings were performed on filter membrane. Following conjugation, the suicide vector pUC18.*abaI*::Tc would be integrated at the wildtype *abaI* gene, that results in the wildtype copy of the *abaI* gene and second copy with the *abaI*::Tc disruption. These exconjugants with both the wildtype and the mutant copy of the *abaI* gene (single crossover event) were selected based on their tetracycline and ampicillin resistance (tetracycline marker was present on the disrupted *abaI* gene and ampicillin marker was on the pUC18 vector). Following this, the exconjugants were grown without the antibiotic pressure. This removes the pressure to maintain the plasmid integration. Then the exconjugants that carry only the mutant *abaI* gene (double crossover event) were selected by eliminating any bacteria that have maintained the plasmid integration and selecting the bacteria that does not grow in the presence of the vector marker (ampicillin). The mutation was confirmed by PCR to get wildtype PCR products followed by sequencing. The *abaI*::Tc mutants failed to produce any AHL signals and were further evaluated for their biofilm forming capabilities and compared with the biofilm formation in the wild type *abaI* gene. The biofilm formation was done as described previously in microtiter plate. To demonstrate that the biofilm inhibition in the mutant was due to the loss of the AHL signal, mutation complementation experiment with the exogenously added AHLs as the ethyl-acetate extracts of the culture supernatants from the wildtype and the mutants was performed. Only ethyl-acetate was added in the negative control samples.
